# 705 Evaluation of Invasive Fungal Infections in the Burn ICU

**DOI:** 10.1093/jbcr/irad045.181

**Published:** 2023-05-15

**Authors:** Sam Pournezhad, Elaina Etter, Rohit Mittal

**Affiliations:** Grady Health System, Atlanta, Georgia; Grady Health System, Atlanta, Georgia; Grady/ Emory, Atlanta, Georgia; Grady Health System, Atlanta, Georgia; Grady Health System, Atlanta, Georgia; Grady/ Emory, Atlanta, Georgia; Grady Health System, Atlanta, Georgia; Grady Health System, Atlanta, Georgia; Grady/ Emory, Atlanta, Georgia

## Abstract

**Introduction:**

Fungal infections are increasingly associated with critical illness, especially in major burn injury. The risk factors of invasive fungal infections include central venous catheter (CVC) placement, mechanical ventilation, broad-spectrum antibiotics, renal replacement therapy (RRT), and total parental nutrition. Critically ill burn patients have additional risk factors including extensive wounds, impaired immune system, and repeated surgical intervention. Despite significant morbidity and mortality caused by invasive fungal infections, efforts to prevent them with antifungal prophylaxis have not improved outcomes. In patients who develop invasive fungal infection, appropriate empiric antifungal therapy is imperative to reduce morbidity and mortality especially in the setting of delayed culture and sensitivity data. The purpose of this study was to characterize risk factors associated with the development of invasive fungal infection, invasive fungal infection organisms and surgical and pharmacological management.

**Methods:**

A retrospective chart review was completed of adult patients admitted to the burn ICU found to have an invasive fungal infection (defined as at least one positive blood or tissue fungal infection and receipt of systemic antifungal therapy). The primary outcome was to identify common fungal organisms. Secondary outcomes included susceptibility pattern of the organism, location of infection, surgical management including debridement and amputation, pharmacological management, median ICU and hospital length of stay, and in hospital mortality.

**Results:**

A total of 10 patients of a goal of 100 patients with a median 54% TBSA burns were evaluated at this time. The most common yeast species included candida albicans; mold species included a variety of organisms such as fusarium, zygomycete and paecilomyces. Common risk factors among these patients included CVC access (57%), mechanical ventilation (43%), and RRT (43%). Yeast infections were primarily treated with anidulafungin and fluconazole, while mold infections were managed with systemic amphotericin as backbone in addition to voriconazole or isavuconazole. Patients underwent aggressive surgical debridement for source control. Median ICU stay was 53 days with a median hospital stay of 63 days, and 42% mortality.

**Conclusions:**

Fungal infections among critically ill burn patients are associated with the high morbidity and mortality rates. Therefore, mitigation of modifiable risk factors is of utmost importance. This retrospective review of common fungal growth in our unit will inform future empiric antifungal management for patients with concern of invasive fungal infections.

**Applicability of Research to Practice:**

Fungal infections present a clinical challenge for burn practitioners, especially in geographically moist areas. Prevention and informed surgical and pharmacological management are key to overcoming this infectious disease threat.

